# Thin-Film Reference Electrodes for Fast-Scan Cyclic Voltammetry

**DOI:** 10.1021/acschemneuro.5c00397

**Published:** 2025-11-19

**Authors:** Yongli Qi, Jaehyeon Ryu, Dongyeol Jang, Bella Schaub, Yieljae Shin, Tianyu Bai, Gen Li, Joshua P. Aronson, James C. Leiter, Hui Fang

**Affiliations:** † Thayer School of Engineering, 3728 Dartmouth College, Hanover, New Hampshire 03755, United States; ‡ 1859 Beth Israel Deaconess Medical Center, Boston, Massachusetts 02215, United States; § White River Junction VA Medical Center, White River Junction, Vermont 05001, United States

**Keywords:** Thin-film, reference electrode, fast-scan cyclic voltammetry, electrochemical sensor, neurotransmitters

## Abstract

Electrochemical sensors rely on reference electrodes (REs) to provide stable potential standards, ensuring accurate and reliable detection. The development of biocompatible, stable, and miniaturized REs to replace conventional Ag/AgCl electrodes is crucial for translating electrochemical sensing for human applications. This study evaluates the performance of thin-film electrodes made from gold (Au), platinum (Pt), poly­(3,4-ethylenedioxythiophene)-polystyrenesulfonate (PEDOT:PSS), and platinum–iridium (Pt–Ir) as REs for fast-scan cyclic voltammetry (FSCV), a widely used technique for real-time neurotransmitter detection. Using dopamine (DA) sensing as a model platform, our results demonstrate that Pt–Ir electrodes provide the necessary stable potential, low drift, and high reproducibility for FSCV sensing, even at a reduced size of 0.1 mm × 0.1 mm. Additionally, Pt–Ir exhibited performance comparable to Ag/AgCl electrodes across various pH levels and in the presence of biofouling agents. These findings highlight Pt–Ir as a promising alternative RE, with strong potential for integration into miniaturized electrochemical sensors for both preclinical and clinical applications.

## Graphical abstract

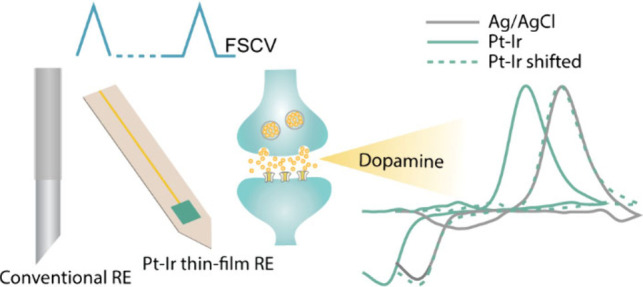

Electrochemical sensors are essential tools for basic science studies and emerging biomedical applications. For accurate and reliable electrochemical measurements, a reference electrode (RE) with good stability is essential across various techniques, including potentiometry, amperometry, and voltammetry.^1^ Fast-scan cyclic voltammetry (FSCV), a widely used voltammetry method, enables real-time monitoring of neurotransmitter dynamics with high temporal resolution and sensitivity and offers high translational potential.^2,3^ FSCV has been widely used for decades as a neurochemical tool for in vivo detection of rapid neurotransmitter fluctuations in animal models. More recently, research efforts have expanded toward human neurochemical studies, with increasing interest in translating FSCV for clinical applications. As a background-subtracted technique, FSCV relies particularly on a stable RE potential to isolate the faradaic current of the target analyte.^4^ However, the REs commonly used in animal studies are Ag wires coated with AgCl, which are cytotoxic, making them unsuitable for human use.^5^ To address these safety concerns, stainless steel (SS) cannulas have been used as the RE for human applications.^6,7^ However, SS is highly polarizable, and its lack of well-defined redox couple and inhomogeneous grain structure can lead to potential instability, compromising measurement sensitivity and specificity.^8^ Thus, there is a critical need for preclinical investigation and validation of novel REs that are both biocompatible and capable of providing stable reference potentials for human use.

As sensing technology advances, miniaturization and integration have also become essential goals in biosensor development. In particular, thin-film electrodes are becoming increasingly popular in microsystems due to their biocompatibility, high spatial resolution, and ease of microfabrication, making them well-suited for integration into next-generation neurochemical sensing platforms. Despite the tremendous progress in electrochemical sensors over the past decades, REs remain largely unchanged. Thin-film Ag/AgCl REs have been developed for integration into microprobes; however, they suffer from chemical and mechanical instability due to AgCl dissolution and coating delamination.^9^ Electrodeposited Pt–Ir has demonstrated excellent adhesion to metal substrates^10^ and corrosion resistance in chronic in vivo studies.^11,12^ Iridium oxide (IrO_*x*_) emerges as a promising alternative, offering superior biocompatibility, enhanced mechanical stability, and a reduced foreign body response compared to Ag/AgCl. Thin-film IrO_*x*_ has been used as an on-probe reference electrode for constant potential amperometric sensing under stable pH conditions.^13–15^ However, the miniaturization and integration of thin-film REs for voltammetric applications remain largely unexplored, posing a major barrier to the clinical translation of implantable electrochemical sensors.

In this work, we studied various thin-film macro/microelectrodes made of Au, Pt, PEDOT:PSS, and Pt–Ir for use as REs in FSCV sensing (Figure 1A). The potential stability of the microfabricated thin-film electrodes was assessed by monitoring their open circuit potentials (OCPs), while their performance as FSCV REs was tested using dopamine (DA) as the analyte. Additionally, the effects of pH and biofouling on the reliability of Pt–Ir electrodes were specifically examined in comparison to conventional Ag/AgCl REs. Our results reveal that thin-film Pt–Ir electrodes are promising candidates to serve as alternative REs for FSCV applications. With superior biocompatibility and miniaturization ability over Ag/AgCl, thin-film Pt–Ir REs possess high potential for practical applications in integrated electrochemical sensors and translational research.

**Figure 1:**
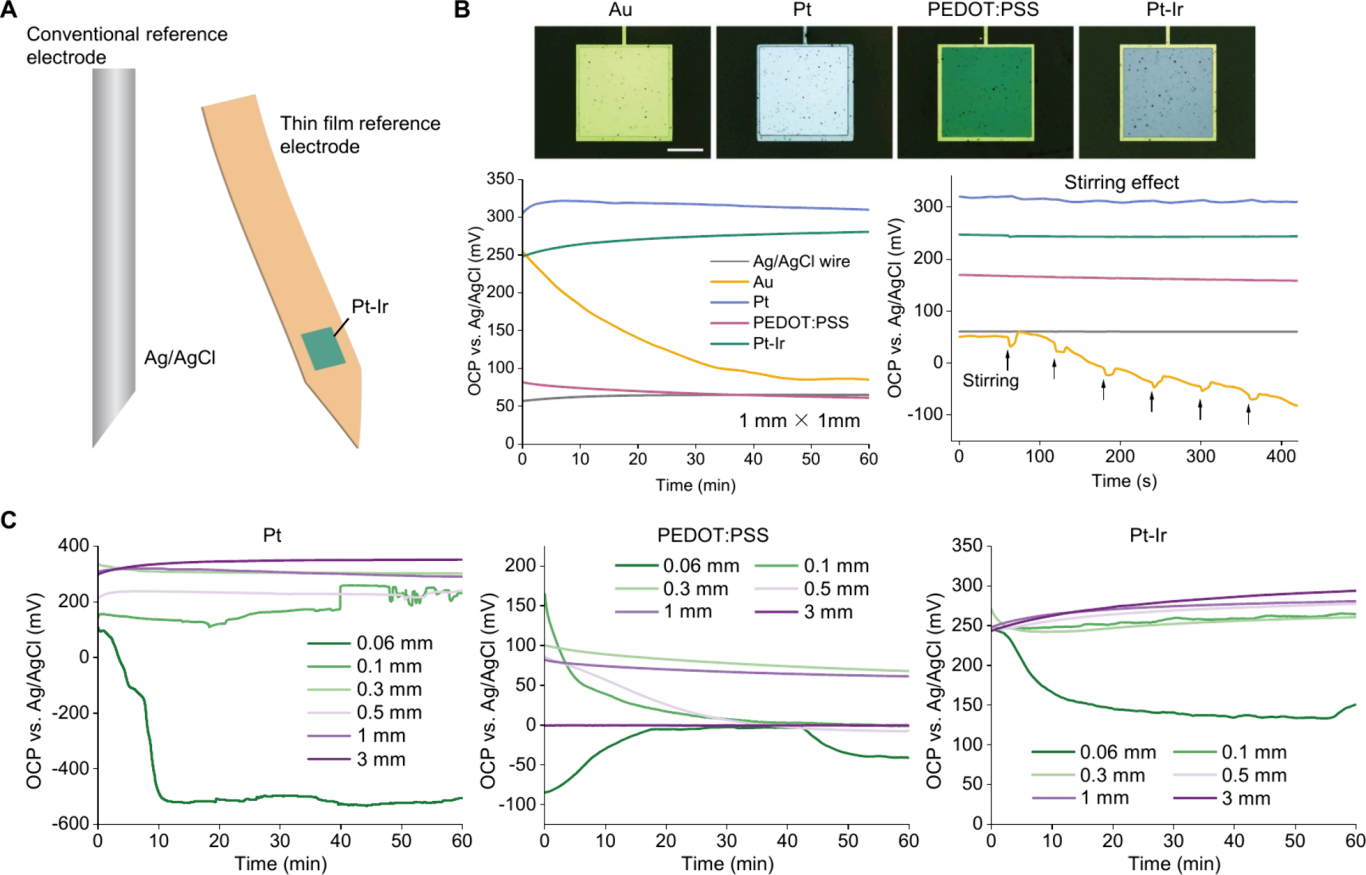
Optical microscope images and OCPs of different thin-film electrodes. (A) Illustration of conventional RE and Pt–Ir thin film RE. (B) Optical microscope images (Top, scale bar: 200 μm), 1-h measurement of OCPs (bottom left), and the effect of stirring on the OCPs (bottom right) for different thin-film electrodes. (C) Comparison of the size dependency on the stability of OCPs.

## Potential Stability of Thin-Film REs

We successfully fabricated thin-film Au and Pt electrodes on 1-mil-thick Kapton using the standard photolithographic method, which allows a patterning resolution down to microns. Additionally, PEDOT:PSS and Pt–Ir electrodes were fabricated by electroplating the coatings onto Au electrodes. The detailed fabrication process is described in Materials and Methods. Optical microscope images confirm that all the thin-film electrodes exhibit uniform surfaces and well-defined dimensions (Figure 1B). Given that potential stability is a key factor for REs, we first measured the OCP of all four types of thin-film electrodes against a commercial glass Ag/AgCl (3 M NaCl) RE. The OCPs were continuously monitored in PBS for 1h, while a Ag/AgCl wire was tested as a control. The Ag/AgCl wire was prepared using the same method and size as the RE commonly used for FSCV in animal studies, with an area of about 0.85 mm^2^. In translational studies, the time allocated for FSCV experimental procedures is limited. For patient safety, the use of FSCV in research must add no more than 15–60 min to the overall procedure in the operating room. Therefore, it is crucial for the REs to quickly reach a stable potential. The OCP of Pt, PEDOT:PSS, and Pt–Ir stabilized more quickly than Au, indicating shorter conditioning times (Figure 1B). An ideal RE should maintain a highly stable potential with minimal drift over time, ideally less than 1 mV/min.^16^ We assessed the potential stability after 10 min of conditioning and found that the potential drift rate of Au is 1.85 mV/min, whereas Pt, PEDOT:PSS, and Pt–Ir electrodes exhibited significantly lower drifts of 0.22, 0.25, and 0.30 mV/min, respectively. Furthermore, these electrodes demonstrated greater stability under stirring conditions compared to Au electrodes (Figure 1B), highlighting their potential as promising RE candidates. The instability of the OCP of gold and its greater susceptibility to stirring, compared to Pt and Pt–Ir, may result from the longer time required for the gold surface to form a stable electrical double layer. In contrast, the oxide layers^17,18^ formed on the surface of Pt and Pt–Ir provides a faradaic charge transfer mechanism, which supports their more stable potential.

To evaluate the reproducibility of the electrode potential while downsizing, we monitored the OCP of Pt, PEDOT:PSS, and Pt–Ir electrodes across a range of sizes (0.06 mm × 0.06 mm to 3 mm × 3 mm). Among them, Pt–Ir exhibited the most consistent and reproducible potential, maintaining stability even at 0.1 mm × 0.1 mm (Figure 1C). Furthermore, OCP measurements of Pt–Ir electrodes with the same size of 1 mm × 1 mm (n = 4) showed minimal variation between samples, further confirming their reproducibility (Figure S1). In comparison, we tested a handmade Ag wire, commonly used in animal studies, with a diameter of 0.254 mm and an exposed chlorinated tip measuring 0.5–1 mm in length. The estimated surface area of the Ag/AgCl wire is comparable to that of a 1 mm × 1 mm thin-film electrode (Figure S2). The stability of the smaller size of Pt–Ir enables the realization of miniaturized electrochemical sensor designs. Overall, Pt–Ir demonstrated excellent potential stability, low drift, and reliable potential retention, even at small RE surface areas. The low potential drift in a reference electrode is essential for ensuring accurate and stable electrochemical measurements, especially in implantable or continuous monitoring applications.

## Performance of Thin-Film REs for FSCV Sensing

We evaluated the performance of Pt–Ir electrodes as a RE for FSCV sensing. DA was used as the model analyte due to its critical role in the control of brain function and its association with a broad spectrum of brain disorders, including Parkinson’s disease and depression.^19^ The carbon sensor used in this study had a size of 0.06 mm × 0.06 mm unless otherwise noted, and this size is comparable to a conventional carbon fiber electrode (0.01 mm in diameter and 0.1 mm in length). Given the potential difference between Pt–Ir and Ag/AgCl, we applied a −0.212 V offset to the FSCV scanning window, calculated based on the position of Faradaic peaks within the background current.

The background voltammogram obtained using Pt–Ir was nearly identical to that of Ag/AgCl RE (Figure 2A), demonstrating the electrochemical stability of Pt–Ir under FSCV measurement. Additionally, after shifting back 0.212 V, the background-subtracted cyclic voltammogram for 500-nM DA closely overlapped that obtained using Ag/AgCl, indicating that the adjustment of the scanning window did not affect dopamine sensitivity or the characteristic voltammogram signature (Figure 2B). Furthermore, the current–time traces extracted at the actual oxidation peak potential from the FSCV voltammograms demonstrated comparable response times and sensitivities between Pt–Ir and Ag/AgCl (Figure 2C, Figure S3). These findings suggest that Pt–Ir performs equivalently to Ag/AgCl as a RE for FSCV sensing, making it a promising alternative for applications requiring biocompatibility and seamless device integration.

**Figure 2:**
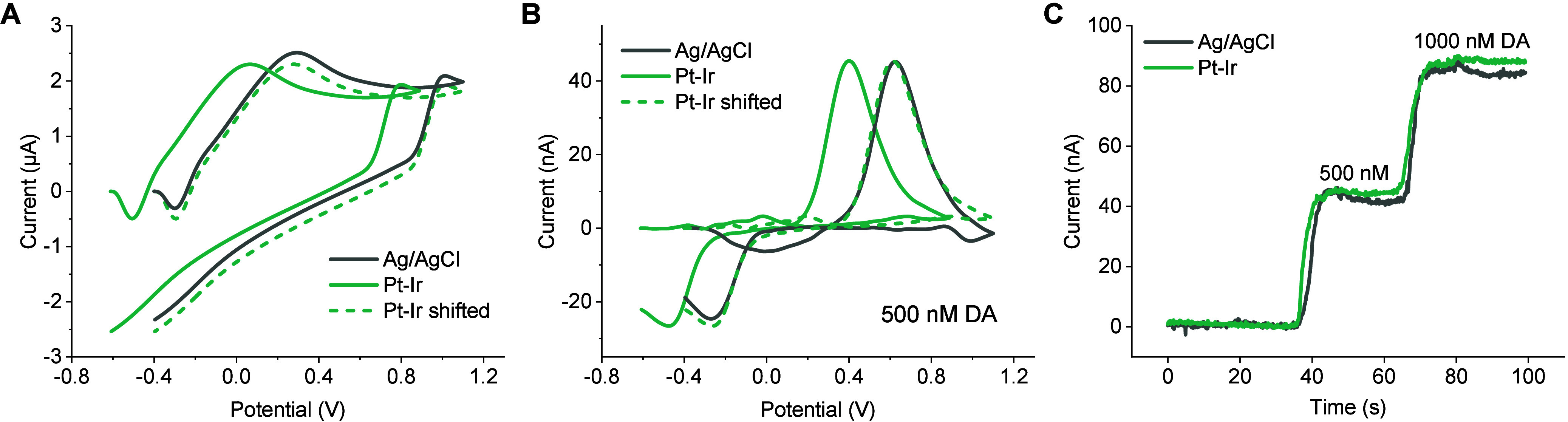
Effect of the potential offset on DA sensing performance using FSCV. Background current (A) and background-subtracted cyclic voltammograms in the presence of 500 nM DA (B) using Ag/AgCl and Pt–Ir (1 mm × 1 mm) as the REs. (C) Current–time traces at the oxidation peak potentials of DA extracted from FSCV data. FSCV was performed at 10 Hz and 400 V/s in PBS (pH = 7.4). A 0.06 mm × 0.06 mm CCM was used as the electrochemical sensor.

Next, we tested all the thin-film electrodes made of Au, Pt, PEDOT:PSS, and Pt–Ir, ranging in size from 3 mm × 3 mm to 0.3 mm × 0.3 mm, for 500-nM DA sensing. Different potential offsets were applied for each type of electrode: −0.072 V for Au, −0.22 V for Pt, −0.012 V for PEDOT:PSS, and −0.212 V for Pt–Ir (Figure 3). For the Au electrode, distortion in the background-subtracted cyclic voltammograms for 500-nM DA sensing was observed starting at a size of 1 mm × 1 mm, and it became more pronounced as the electrode size further decreased. A significant reduction in background current was also noted at 0.3 mm × 0.3 mm size. In comparison, Pt and PEDOT:PSS electrodes exhibited only minor changes in the shape of the background current and background-subtracted cyclic voltammograms, although peak shifts were observed across different electrode sizes. Among all the tested materials, Pt–Ir electrodes exhibited the most consistent background current and background-subtracted cyclic voltammograms, indicating excellent reproducibility and stability across different electrode sizes. While PEDOT:PSS remained functional at a size of 0.06 mm × 0.06 mm, size-dependent offsets were required (Figure S4). Given that the high electrochemically active surface area of PEDOT:PSS allows for a smaller geometric area ratio between the reference and working electrodes, improving the consistency of its reference potential across samples will be critical to establishing PEDOT:PSS as a reliable thin-film reference electrode for FSCV—an advancement that would significantly benefit device miniaturization. The observed stability in FSCV sensing aligns with the OCP results and tends to correlate with the impedance of each electrode, where lower impedance corresponds to more consistent FSCV voltammograms as electrode size decreases (Figure S5). This finding is consistent with the standard requirements for a reference electrode to have low impedance.^20^

**Figure 3:**
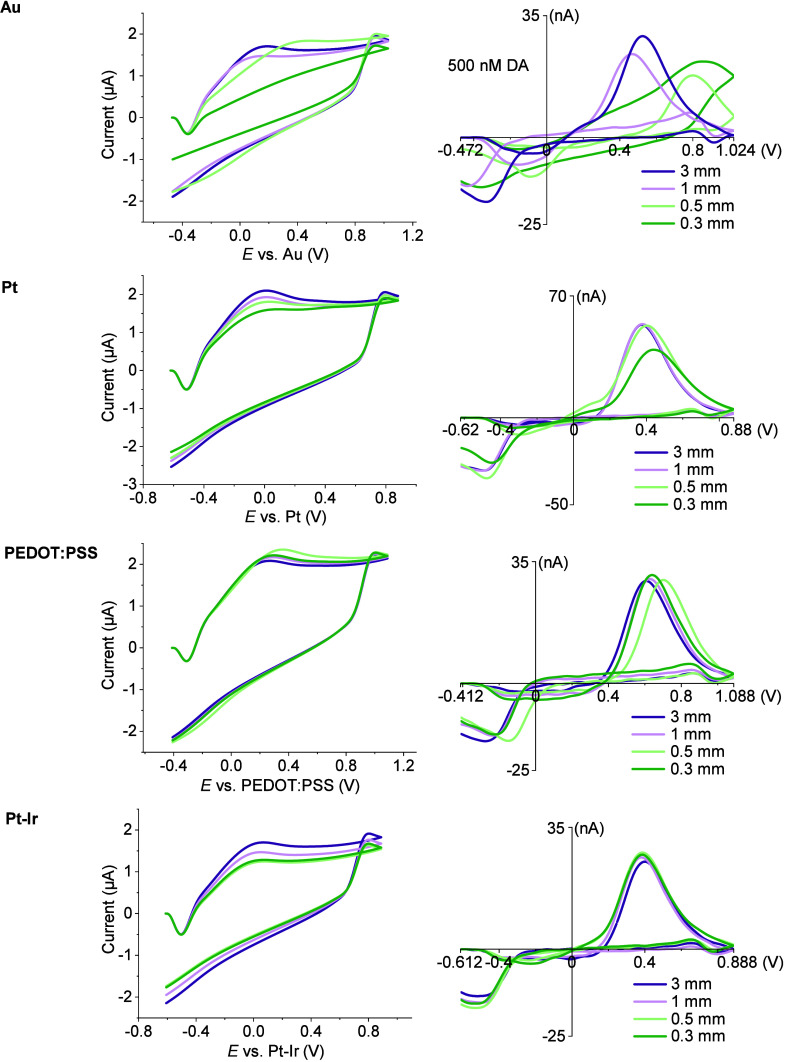
Comparison of DA sensing performance with different thin-film REs. Background current (left) and background-subtracted cyclic voltammograms for 500 nM DA (right) with a RE size ranging from 3 mm × 3 mm to 0.3 mm × 0.3 mm.

Notably, when using a 0.06 mm × 0.06 mm carbon sensor, the 0.3 mm × 0.3 mm Pt–Ir thin-film RE produced an identical DA sensing curve to the 3 mm × 3 mm electrode. When the carbon sensor was reduced to 0.03 mm × 0.03 mm, a 0.1 mm × 0.1 mm Pt–Ir RE maintained comparable performance to the commercial Ag/AgCl RE (Figure S6). These findings indicate that the ratio between the Pt–Ir RE and carbon sensor areas plays a crucial role in sensing performance. Typically, this ratio should exceed 10 to prevent distortion of the background-subtracted cyclic voltammogram for DA. Miniaturization of the carbon sensor is necessary when using Pt–Ir as the reference electrode to achieve a compact neural probe. For example, a 10 μm × 10 μm carbon sensor enables the use of Pt–Ir reference electrodes as small as 34 μm × 34 μm while still maintaining reliable reference performance. These results suggest the potential for integrating high-density carbon sensors with a miniaturized thin-film RE to enable compact device designs.

## Effect of pH on the Performance of Pt–Ir REs

A potential issue associated with alternative REs is their sensitivity to hydrogen ions, meaning that changes in the pH of the test solution can influence the OCP of this RE. We evaluated the pH response of all four types of thin-film electrodes, and Pt–Ir exhibited a response of −48 mV per pH unit (Figure S7). To further assess the impact of pH on Pt–Ir when used as a RE for FSCV, we compared the background-subtracted cyclic voltammograms of 500-nM DA across a pH range from 2.0 to 10.0. The results indicated that Pt–Ir performed similarly to Ag/AgCl as a RE across the pH levels near the physiological range (pH 4.0–8.5) (Figure 4A).

**Figure 4:**
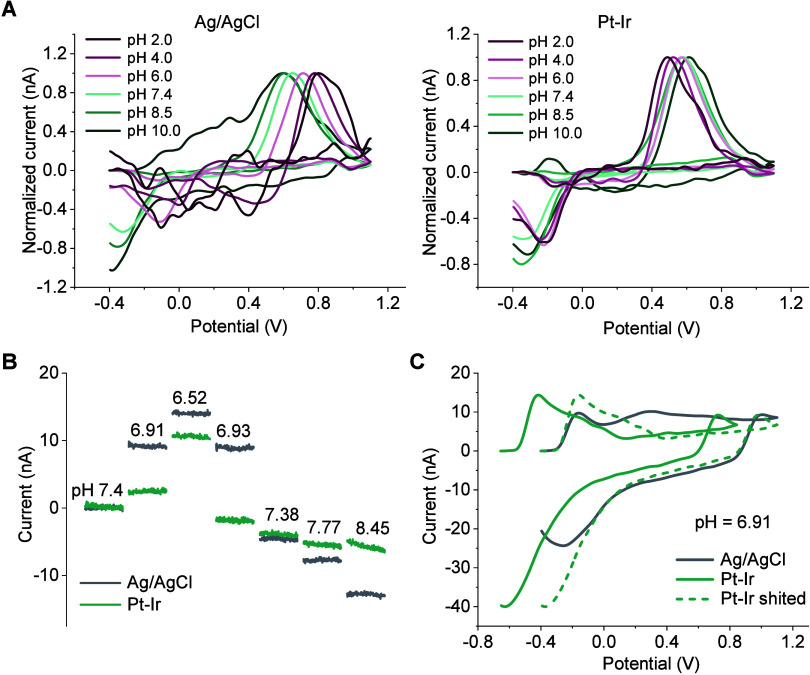
Effect of pH on DA sensing performance using Pt–Ir as the RE. (A) Comparison of background-subtracted cyclic voltammograms for 500 nM DA in different pH using Ag/AgCl (left) and Pt–Ir (right) as RE. Current values were normalized by dividing the peak current at oxidation peak potentials. (B) Changes in current at oxidation peak potentials of DA with varying pH using Ag/AgCl and Pt–Ir as RE. (C) Background-subtracted voltammograms at pH 6.91, referenced to pH 7.4. The Pt–Ir measured 1 mm × 1 mm, while the Ag/AgCl had a diameter of 2 mm and a length of 4 mm.

Since the current–time trace at the oxidation peak potential is commonly used as an indicator of dopamine concentration changes, we analyzed the current at 0.6 V collected while continuously varying the pH. A smaller current change was observed in the Pt–Ir group compared to Ag/AgCl (Figure 4B). The background-subtracted cyclic voltammograms retained similar shapes for both Pt–Ir and Ag/AgCl when the pH decreased from 7.4 to 6.91, with Pt–Ir showing a smaller current variation around 0.6 V (Figure 4C). This indicates that pH changes may cause less interference to DA sensing when using Pt–Ir. The current change observed at 0.6 V is induced by pH-dependent background current variations, which are influenced by the double-layer capacitance and redox reactions involving electrochemically active surface groups of the carbon sensor.^21^ While the potential of the Ag/AgCl electrode remains stable across different pH levels (Figure S8), the potential of the Pt–Ir electrode can shift with pH. This potential shift may partially offset the background current change caused by variations in pH, resulting in a smaller current change at 0.6 V. Overall, Pt–Ir exhibited performance comparable to, or even better than, Ag/AgCl under pH fluctuations. Moreover, the fairly regulated pH in vivo, particularly in the brain,^22^ provides an environment for stable Pt–Ir RE potential. Thus, in vivo applications of Pt–Ir are expected to experience minimal background current variations caused by pH changes. Our electroplated IrO_*x*_ exhibited a Nernstian response of approximately −59 mV/pH (Figure S9), consistent with literature reports.^23^

As a candidate material for thin-film reference electrodes in electrochemical sensors, IrO_*x*_ was also evaluated by electroplating an IrO_*x*_ coating onto microfabricated gold electrodes for comparison. The IrO_*x*_ REs exhibited impedance similar to that of Pt–Ir (Figure S5), but showed greater variability in OCP across electrodes of different sizes. Additionally, shifts in the oxidation peak of dopamine were observed when using IrO_*x*_ REs of different sizes as the reference (Figure S10). These inconsistencies may be attributed to potential drift and the unpredictable electrochemical behavior of hydrous IrO_*x*_ prepared through electroplating.^23^

## Effect of Biofouling

Reference electrode fouling can negatively impact the sensing accuracy. To examine the effects of biofouling on Pt–Ir REs, we tested them in a solution containing bovine serum albumin (BSA), a widely used in vitro standard for biofouling evaluation in electrode and sensor testing.^24–26^ We then evaluated the impact of biofouling on their performance for DA sensing. Nafion-coated Pt–Ir and Ag/AgCl wire were used as control groups. After soaking in PBS containing 40 mg/mL BSA at 37 °C for 7 days, no degradation in the electrochemical signature of DA was observed, although a different offset potential was required to reproduce the same background-subtracted cyclic voltammograms (Figure 5A,B). For both Pt–Ir and Nafion-coated Pt–Ir, the reference potential exhibited a drift of approximately 0.3 V after 1 day of soaking. From day 1 to day 7, the potential remained stable, with minor deviations within ± 50 mV—comparable to the variation observed with the Ag/AgCl wire (Figure 5C,D). A similar trend was observed for Pt–Ir soaked in PBS without BSA, suggesting that the initial potential drift may result from anion adsorption on the electrode surface (Figure 5D and Figure S11). Additionally, using Pt–Ir as the reference electrode, we successfully detected dopamine signals in the mouse brain 3 days after implantation (Figure S12). The scanning window was consistent with that used in the day-3 bench biofouling test as shown in Figure 5B.

**Figure 5:**
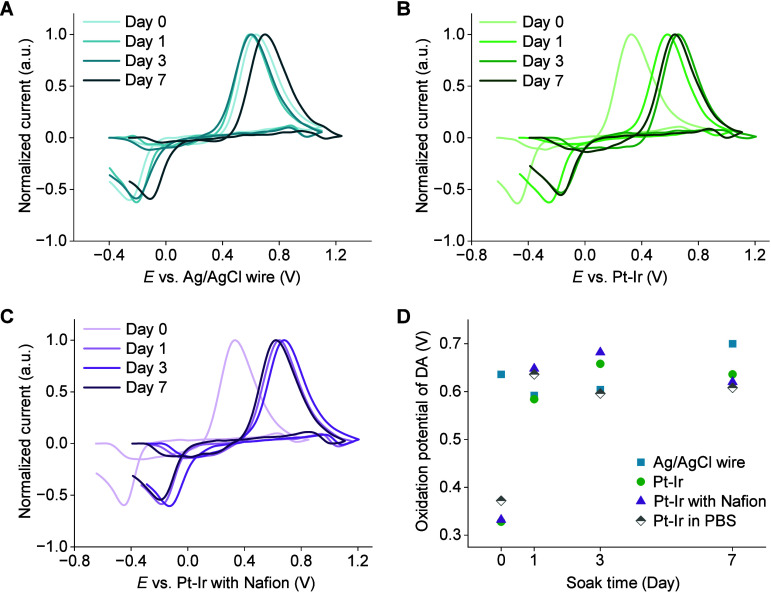
Biofouling assessment in BSA. Background-subtracted cyclic voltammograms of 500 nM DA using (A) Ag/AgCl wire, (B) Pt–Ir, and (C) Nafion-coated Pt–Ir as reference electrodes after soaking in PBS containing 40 mg/mL BSA for the indicated time. Currents were normalized to the peak oxidation current of DA. (D) DA oxidation potential over soak time, used to assess potential shifts in the reference electrodes during the soak test.

Previous studies have reported 200-mV peak voltage shifts in FSCV signals due to the Ag/AgCl electrodes, revealing the biofouling complications related to the REs.^27^ In FSCV signals, this change can be recognized by the position of Faradaic peaks within the background current and compensated by applying a potential offset.^3,28^ These findings confirm that adjusting the scanning window based on the positions of the Faradaic peaks does not affect the sensitivity or the voltammogram signatures. Nafion, a widely used antibiofouling coating in electrochemical sensors, did not compromise the stability of the reference potential and shows promise for mitigating biofouling-induced drift in future in vivo applications. Our soak test results suggest that a 24-h presoak in PBS could help reduce in vivo potential drift of Pt–Ir, potentially keeping it within ± 50 mV. Although Pt–Ir exhibits less potential stability compared to Ag/AgCl, FSCV, as a voltammetric technique, does not require an extremely stable RE potential, unlike potentiometric sensors, where any offset or drift in the RE potential would directly alter the output signal. The superior biocompatibility of Pt–Ir is well established through its decades-long use in FDA-approved deep brain stimulation devices and other neural implants, giving it a clear advantage over Ag/AgCl for in vivo applications.^29–31^

Utilization of the miniaturized on-probe Pt–Ir RE could help reduce tissue damage and noise compared to the utilization of a separate Ag/AgCl RE.

## Conclusions

We explored the potential of photolithographically fabricated thin-film electrodes as REs for FSCV sensing. Among these, Pt–Ir exhibited the most consistent and stable potential with the best electrode size scalability as well. Pt–Ir thin-film REs demonstrated reliable performance in neurotransmitter detection for FSCV applications, maintaining comparable sensing capabilities to Ag/AgCl even under pH fluctuations and biofouling. Moreover, the fabrication process and materials composition of Pt–Ir thin-film electrodes are fully compatible with our recently developed carbon-coated microelectrode (CCM) sensor,^32^ enabling seamless integration of RE and electrochemical sensors into a compact neural probe. This advancement also facilitates the development of multifunctional neuroelectronic devices. Additionally, Pt–Ir offers superior biocompatibility and miniaturization, making it a promising alternative to Ag/AgCl for translational FSCV applications.

While we have demonstrated the stable performance of thin-film Pt–Ir REs in vitro, their behavior in complex biological environments, including potential biofouling, tissue responses, and long-term electrochemical stability, requires further investigation. Prospective antifouling strategies include applying high-protein-resistivity coatings, such as hydrogel and negatively charged polymers, or modifying surface topography by creating micro- and nanostructures. Future work will also focus on in vivo validation of Pt–Ir RE reliability and its key advantages in integrating carbon sensors and RE onto a single microprobe, expected to achieve simplified, minimally invasive surgery, reduced tissue damage, and minimized noise.

## Materials and Methods

### Fabrication of Thin-Film Au Electrodes

The thin-film Au electrodes were constructed by four layers: a Kapton as the substrate, Cr/Au bilayer as the conductor, and SU-8 as an encapsulation layer. We first laminated 1-mil Kapton film (Fralock) onto a glass slide prespin-coated with polydimethylsiloxane (base/cross-linker 10:1, Sylgard 184). Subsequently, we deposited Cr (10 nm)/Au (70 nm) films on the Kapton substrates using an electron-beam (e-beam) evaporator, with Cr serving as an adhesion layer between the Kapton and Au. Photolithography steps were then undertaken to pattern the metallic traces using a positive photoresist (PR, S1805). The unpatterned Cr/Au was removed with Cr and Au etchants (Transene Co., Inc.), and the photoresist layer was removed by swabbing and rinsing with acetone. Finally, an SU-8 encapsulation layer (1.5 μm; SU-8 2, Kayaku Advanced Materials) was spin-coated on the device to define the microelectrodes, and the SU-8 was cured in ambient air at 180 °C for 30 min. Thin-film gold electrodes with a size of 3 mm × 3 mm, 1 mm × 1 mm, 0.5 mm × 0.5 mm, and 0.3 mm × 0.3 mm, 0.1 mm × 0.1 mm and 0.06 mm × 0.06 mm were fabricated on flexible substrates through these processes.

### Preparation of Thin-Film PEDOT:PSS and Pt–Ir Electrodes

PEDOT:PSS and Pt–Ir electrodes were prepared by electroplating the coatings on thin-film gold electrodes fabricated as described above. Electroplating of PEDOT:PSS was performed following a protocol reported in our previous study.^33^ Specifically, a constant current of 0.2 mA·cm^–2^ was applied to the gold electrode for 90 s in galvanostatic mode (Reference 600+, Gamry Instruments, Inc.). We prepared the solution by mixing 0.01 mol/L ethylene dioxythiophene (EDOT) monomer and 0.1 mol/L poly­(styrenesulfonate) sodium salt (NaPSS) in 150 mL deionized water, followed by stirring for 30 min.^34^ Based on a previously reported deposition rate of 1.7 nm/s^33^, the resulting PEDOT:PSS coating is estimated to be 153 nm thick. Pt–Ir was electroplated on the thin-film gold electrodes using cyclic voltammetry method (−0.1 to 0.1 V, 500 mV/s, 700 cycles) in freshly made Pt–Ir precursor solution at 55 to 60 °C.^35^ The precursor solution was prepared by adding 640 μL HNO_3_ (ACS grade, 68–70%), 0.02 g Na_3_IrCl_6_·xH_2_O (Sigma-Aldrich), and 0.0186 g Na_2_PtCl_6_·6H_2_O (Sigma-Aldrich) into 100 mL deionized water. After boiling for 3 min, the solution was cooled to 55 to 60 °C for electroplating. Based on the prior work using the same parameters, the Pt–Ir thickness is estimated to be 153 nm, corresponding to a deposition rate of 16.5 nm/min.^35^ Here, a typical three-electrode configuration was adopted, including Ag/AgCl reference electrode with filling solution of 3 M NaCl (BASi, USA) and Pt as counter electrode. The thin-film Au electrodes were immersed into the monomer or precursor solutions as working electrodes.

### Fabrication of Thin-Film Pt Electrode

Pt REs of varying dimensions were fabricated to assess their performance. The electrodes were constructed by a Kapton substrate, a Ti/Pt metal electrode with interconnections and contact pads, and a SU-8 encapsulation layer. Initially, a 1 mil Kapton film (Fralock) was laminated onto a glass slide precoated with a fully cured 10:1 PDMS layer (∼30 μm; Sylgard 184). This was followed by O_2_ plasma treatment to facilitate surface cleaning and enhance adhesion for subsequent processing. A lift-off photoresist mask (9 μm, NR9-8000, Futurrex) was then patterned via lithography. Next, a 10 nm Ti adhesion layer and a 100 nm Pt layer were deposited using DC-magnetron sputtering following O_2_ plasma treatment. The electrode, interconnection traces, and contact pads were defined through a lift-off process in acetone, followed by rinsing with isopropyl alcohol and deionized water. Following metal patterning, an SU-8 encapsulation layer (3.5 μm; SU-8 2005, Kayaku Advanced Materials) was spin-coated onto the device, leaving designated openings over the RE sites and connection pads. Finally, the encapsulation layer was cured at 180 °C for 30 min in ambient air.

### Open Circuit Potential (OCP) Measurement

The OCP was monitored using a three-electrode system with Pt wire as the counter electrode, an Ag/AgCl electrode (3 M NaCl) as the RE, and thin-film electrodes as the working electrodes in 1 × PBS (pH 7.4) with a potentiostat (Reference 620, Gamry). As a control, an Ag/AgCl wire was tested. This wire was prepared by soaking PFA-coated silver wire (Dia. 0.254 mm, Catalog# 787000, A-M systems, WA) with an exposed tip of 0.5–1 mm in bleach (Clorox, Oakland, CA) overnight. This approach is a simple and commonly used method in which sodium hypochlorite oxidizes the silver wire surface, forming an AgCl layer at the tip. The stirring effect was tested by stirring the PBS for 10 s every minute using different thin-film electrodes of 1 mm × 1 mm.

### Optical Microscope Images

An optical microscope (Olympus) was used to take sample images and check uniformities across the whole sample of 0.5 mm × 0.5 mm thin-film REs.

### Dopamine Sensing Performance Using Thin-Film Reference Electrodes for FSCV

To evaluate the suitability of thin-film REs for real-world applications, dopamine sensing was performed. Our recently developed carbon-coated microelectrode (CCM)^32^ was used as the electrochemical sensor for FSCV measurements. The CCM was fabricated by potentiostatic electrodeposition of a carbon coating onto 30 × 30 μm^2^ gold microelectrodes, which were fabricated as described above. To confine the deposition area, another layer of 1.5 μm positive photoresist (PR, S1813, Dow) with a 60 × 60 μm^2^ opening was aligned to the underlaying 30 × 30 μm^2^ gold microelectrode. The electrodeposition of carbon coating was performed in a 1 mg/mL graphene oxide (GO) dispersion using a standard three-electrode setup, with the gold microelectrode as the working electrode, Ag/AgCl (3 M NaCl) as the reference electrode, and a Pt wire as the counter electrode. The GO dispersion was obtained by diluting a 4 mg/mL commercial GO dispersion (Graphenea, Spain) with PBS (1×, pH = 7.4) in a 1:3 ratio. Prior to deposition, the gold microelectrodes were treated with 10 min UVO (UV/Ozone ProCleanerTM, Bioforce Nanosciences) to clean the surface and enhance hydrophilicity. Using a potentiostat (Gamry Instruments, Warminster, PA), a constant potential of −0.8 V was first applied for 5 min to deoxygenate the GO solution, followed by electrodeposition at −1.0 V for 3–5 min. During deposition, the dispersion was mechanically stirred at approximately 70 rpm. Afterward, the electrodes were left at room temperature overnight. The PR layer was then removed with acetone, and the devices were annealed at 250 °C for 1 h under an N_2_ atmosphere (Eurotherm furnace, Thermo Scientific), resulting in a 60 × 60 μm^2^ CCM (Figure S13).

Thin-film Au, Pt, PEDOT:PSS, and Pt–Ir electrodes with a size of 3 mm × 3 mm, 1 mm × 1 mm, 0.5 mm × 0.5 mm, and 0.3 mm × 0.3 mm, respectively, were tested as REs. Before neurotransmitter sensing, all CCMs were electrochemically conditioned in PBS to stabilize the background current using a triangular waveform. The FSCV was performed using High-Definition Cyclic Voltammetry software (HDCV, University of North Carolina at Chapel Hill) in conjunction with data acquisition cards (National Instruments, Austin, TX) to control and record data from a potentiostat (WaveNeuro, Pine Research). The WaveNeuro works in a two-electrode configuration with the counter and reference electrode shorted. A triangular waveform with a scan rate of 400 V/s at 10 Hz was applied to a CCM for DA (≥99%, Sigma-Aldrich) sensing. A 1 mM DA solution was prepared in PBS and added into blank PBS in specific volume to achieve a 500 nM concentration step. When using Ag/AgCl as the RE (2.0 × 4 mm, World Precision Instrument), the scanning window ranged from −0.4 to 1.1 V. For thin-film REs, an offset was applied based on the Faradaic peaks in the background current, resulting in the following scanning windows: Au (−0.472 to 1.028 V), Pt (−0.62 to 0.88 V), PEDOT:PSS (−0.412 to 1.088 V), and Pt–Ir (−0.612 to 0.888 V). For all FSCV measurements, the Ag/AgCl used is a commercial Ag/AgCl rod (2 mm diameter, 4 mm length, World Precision Instruments).

### Effect of pH on DA Sensing Performance Using a Thin-Film Pt–Ir Reference Electrode

A thin-film Pt–Ir RE (1 mm × 1 mm) was used for 500 nM DA sensing with FSCV. The PBS solution was prepared by dissolving one PBS tablet (Fisher BioReagents) in 200 mL DI water, and then the pH was tuned to 2.0, 4.0, 6.0, 7.4, 8.5, and 10.0 using 1 M HCl or 1 M NaOH. Dopamine sensing was then conducted in PBS at each adjusted pH. For quasi-dynamic pH effect testing, the CCM and RE were initially scanned in PBS at pH = 7.4. The pH was then gradually decreased to 6.52 and subsequently increased to 8.45 by adding specific volumes of 1 M HCl or 1 M NaOH. Current–time traces at dopamine peak oxidation potentials were extracted to evaluate the effect of pH on baseline currents. Background-subtracted voltammograms for pH 6.91 were obtained by subtracting the background current at pH 7.4. To account for the potential shift with the Pt–Ir RE, an offset of 0.212 V was applied to facilitate voltammograms comparisons. The same tests were conducted using Ag/AgCl RE as a control.

### Biofouling Test

Pt–Ir and Nafion-coated Pt–Ir electrodes (1 mm × 1 mm), along with Ag/AgCl wire, were soaked in PBS containing 40 mg/mL BSA and 0.25 mg/mL NaN_3_ at 37 °C. These electrodes were evaluated as reference electrodes (REs) for FSCV detection of 500 nM DA in PBS on day 0, day 1, day 3, and day 7. The oxidation peak potential of DA was extracted to assess the reference potential shift over time.

## Supplementary Material



## Notes

Impedance, pH response, in vivo validation, and additional dopamine sensing data (PDF)
