# Supplementary material for: Thin-Film Reference Electrodes for Fast-Scan Cyclic Voltammetry
Source: ACS Chem Neurosci. 2025 Nov 19;16(23):4420–8. doi: 10.1021/acschemneuro.5c00397 (PMC12679531; doi:10.1021/acschemneuro.5c00397)
Supplement: Supplementary file 1 [file cn5c00397_si_001.pdf]

Supporting Information

Thin Film Reference Electrodes for Fast-Scan Cyclic Voltammetry

*Yongli Qi[1], Jaehyeon Ryu[1], Dongyeol Jang[1], Bella Schaub[1], Yieljae Shin[1], Tianyu Bai[1], Gen Li[1], Joshua P. Aronson[2], James C. Leiter[3], Hui Fang[1]**

[1]Thayer School of Engineering, Dartmouth College, Hanover, NH, 03755, USA

[2]Beth Israel Deaconess Medical Center, Boston, MA, 02215, USA

[3]White River Junction VA Medical Center, White River Junction, VT, 05001, USA

**Corresponding author. E-mail: hui.fang@dartmouth.edu*

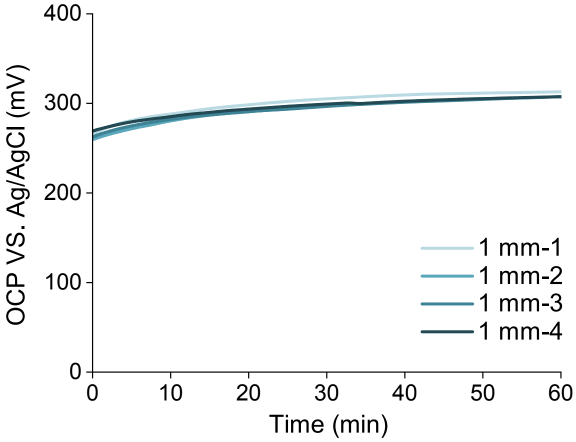

Figure S1. Open-circuit potential across four Pt-Ir electrodes of the same size (1 mm × 1mm).

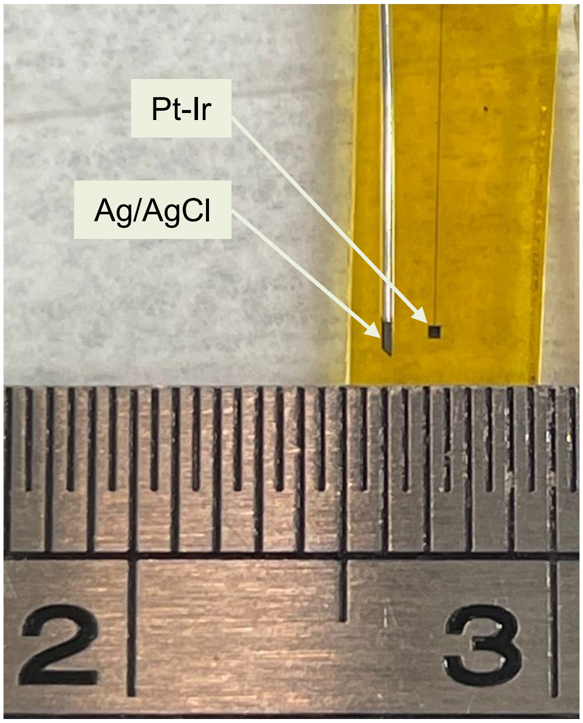

Figure S2. Photograph of conventional Ag/AgCl wire reference electrode and a thin-film Pt-Ir electrode (300 μm × 300 μm). The Ag/AgCl wire has a diameter of 254 μm and an exposed chlorinated tip about 1 mm in length. The Ag wire is encapsulated in PFA (A-M system), resulting in an overall coated diameter of 330.2 μm.

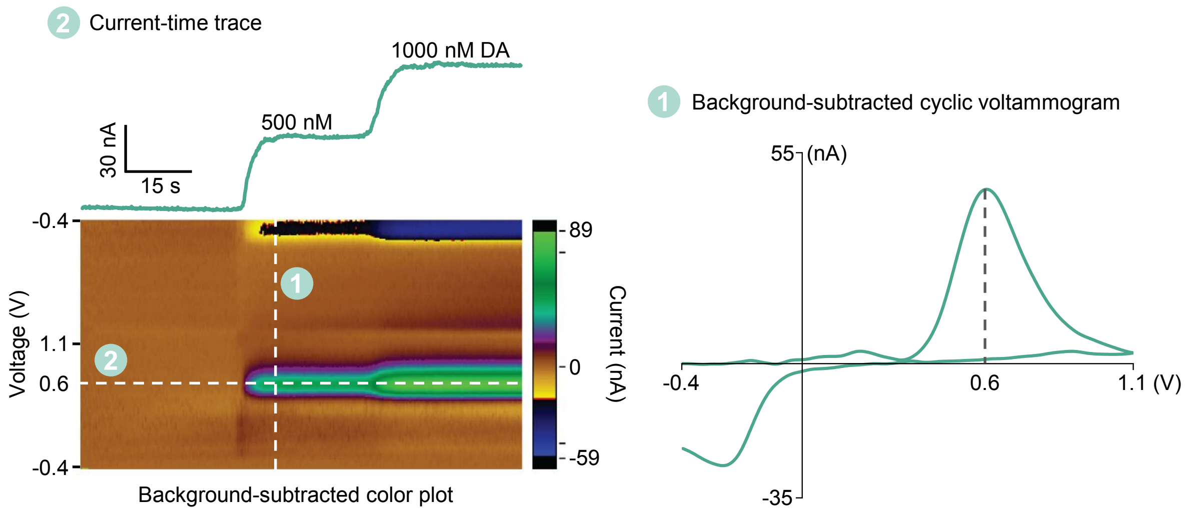

Figure S3. Illustration of FSCV data representation. The plot consists of a continuous stack of background-subtracted cyclic voltammograms over time. A complete background-subtracted cyclic voltammogram (right, ①) can be obtained by taking a vertical slice through the color plot to examine current changes across different voltages. The current-time trace (top, ②) at the oxidation potential for dopamine (0.6 V) can be generated by taking a horizontal slice through the color plot, providing a clear visualization of dopamine changes over time.

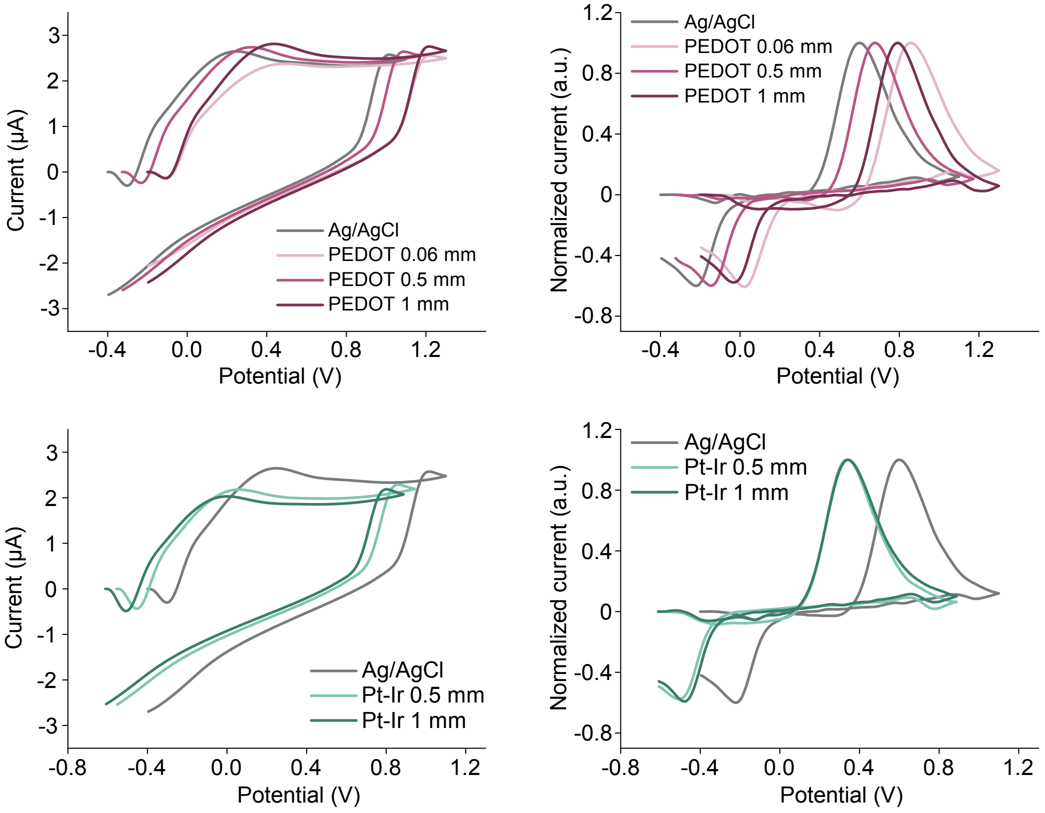

Figure S4. Effect of electrode size on FSCV sensing using PEDOT:PSS and Pt-Ir as reference electrodes. Background current (left) and normalized background-subtracted cyclic voltammograms for 500 nM DA sensing (right). FSCV was performed at 400 V/s and 10 Hz.

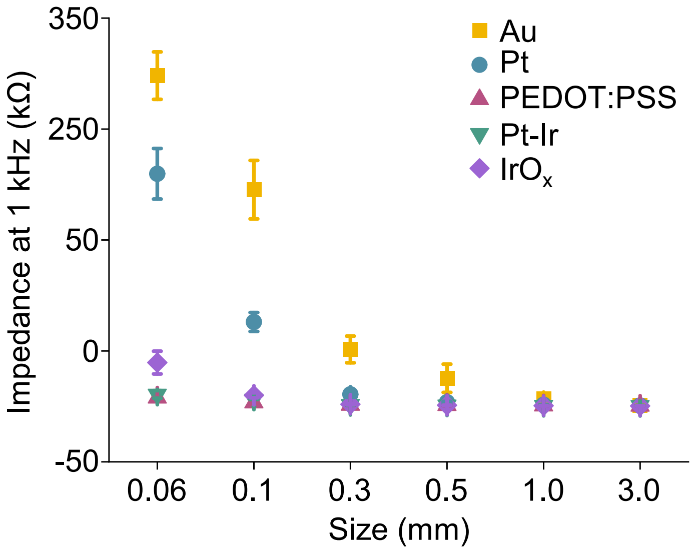

Figure S5. Impedance (at 1 kHz) of the five different types of thin-film electrodes of varying sizes (n = 4 electrodes). Measurement was performed in PBS (pH = 7.4).

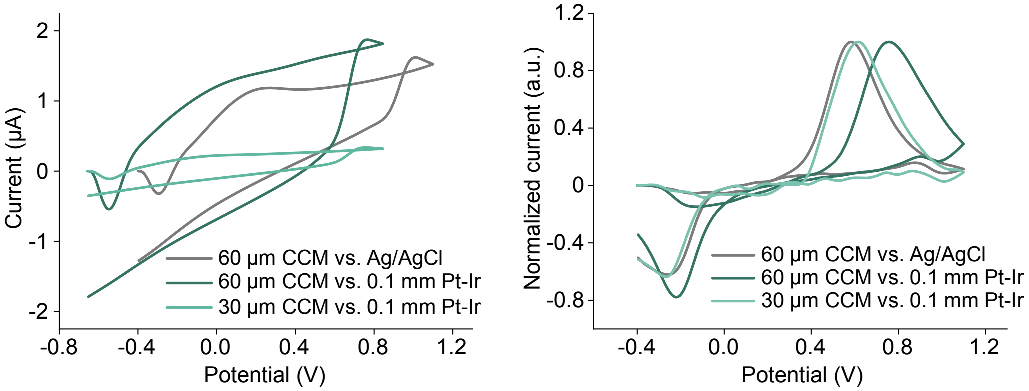

Figure S6. Effect of CCM size on the sensing performance with a Pt-Ir reference electrode. Background current (left) and normalized background-subtracted cyclic voltammograms for 500 nM DA (right). With a 100 µm × 100 µm Pt-Ir reference electrode, the 60 µm × 60 µm CCM exhibits changes in voltammogram signature, while the 30 µm × 30 µm CCM produces a voltammogram nearly identical to that obtained with Ag/AgCl.

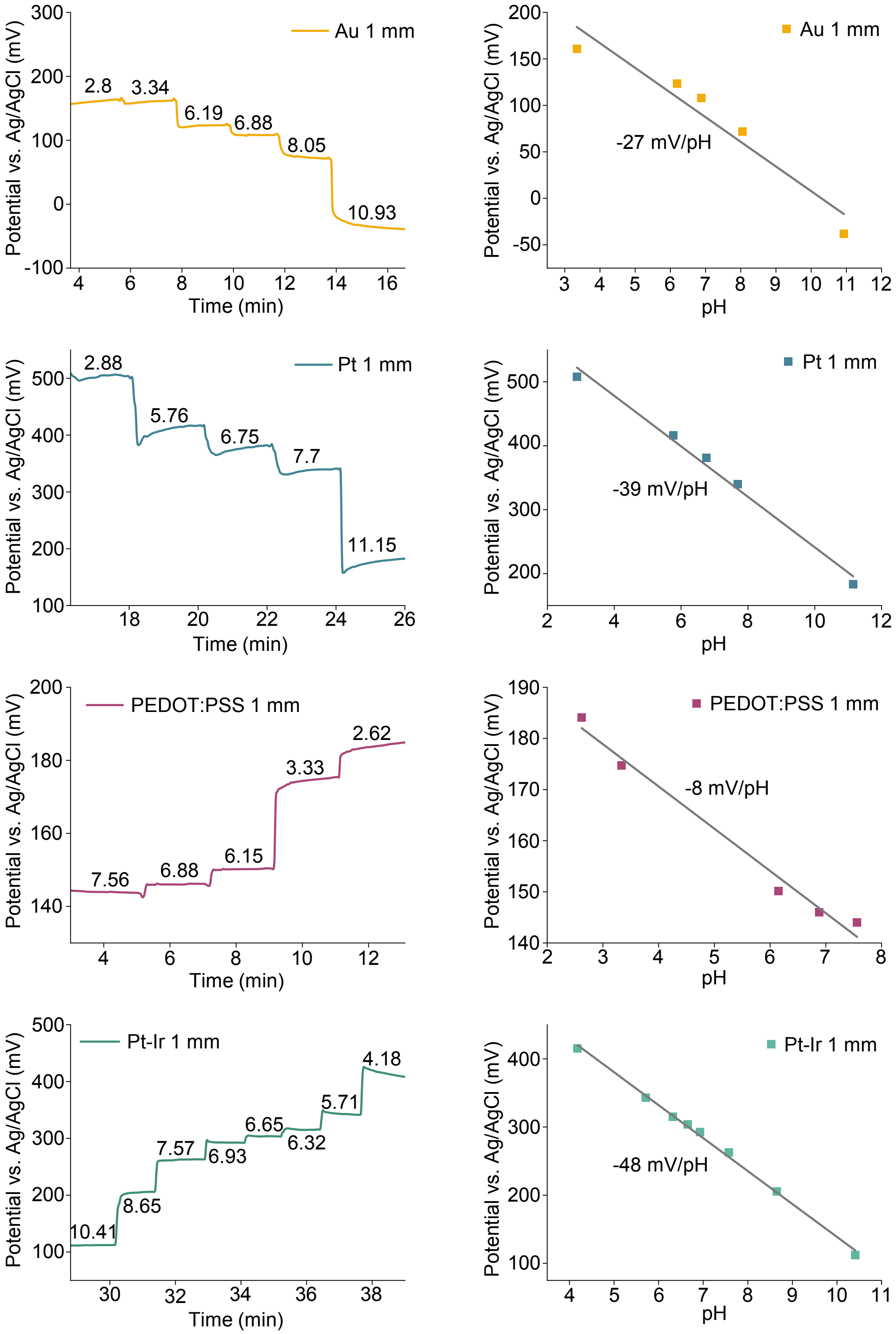

Figure S7. Dynamic step response of open circuit potential to pH changes of four types of thin-film electrodes, referenced to Ag/AgCl (3M NaCl).

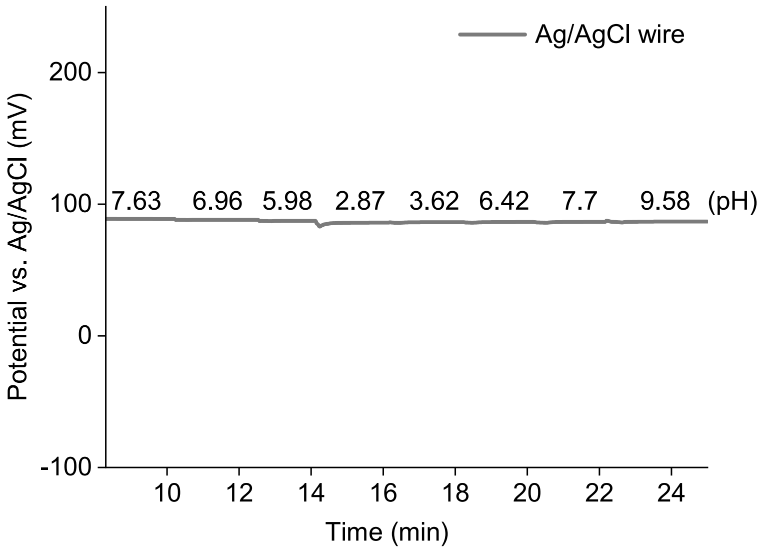

Figure S8. Dynamic step response of open circuit potential to pH changes of Ag/AgCl wire, referenced to Ag/AgCl (3M NaCl).

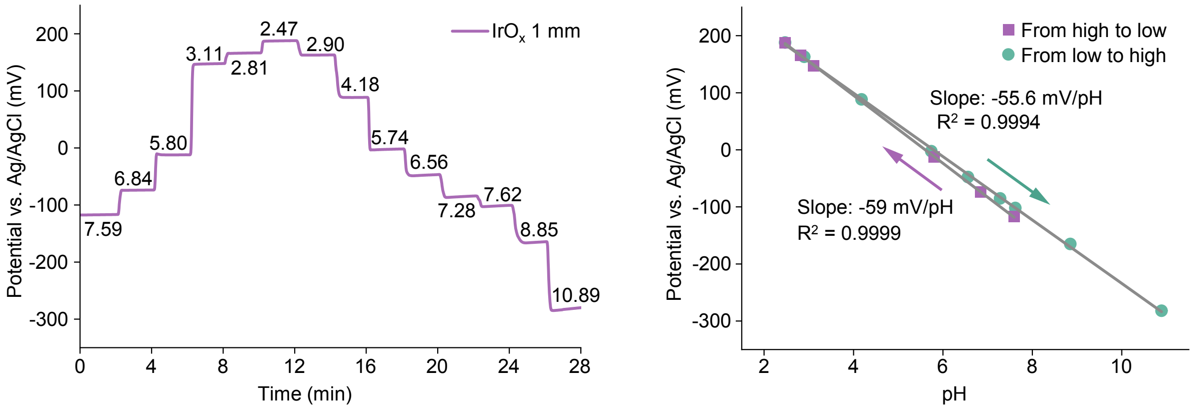

Figure S9. Dynamic step response of the open-circuit potential to pH changes (left) and the linear correlation between potential and pH changes (right) for the electrodeposited thin-film IrO_x electrode, referenced to Ag/AgCl (3 M NaCl). The size of the IrO_x electrode is 1 mm × 1 mm.

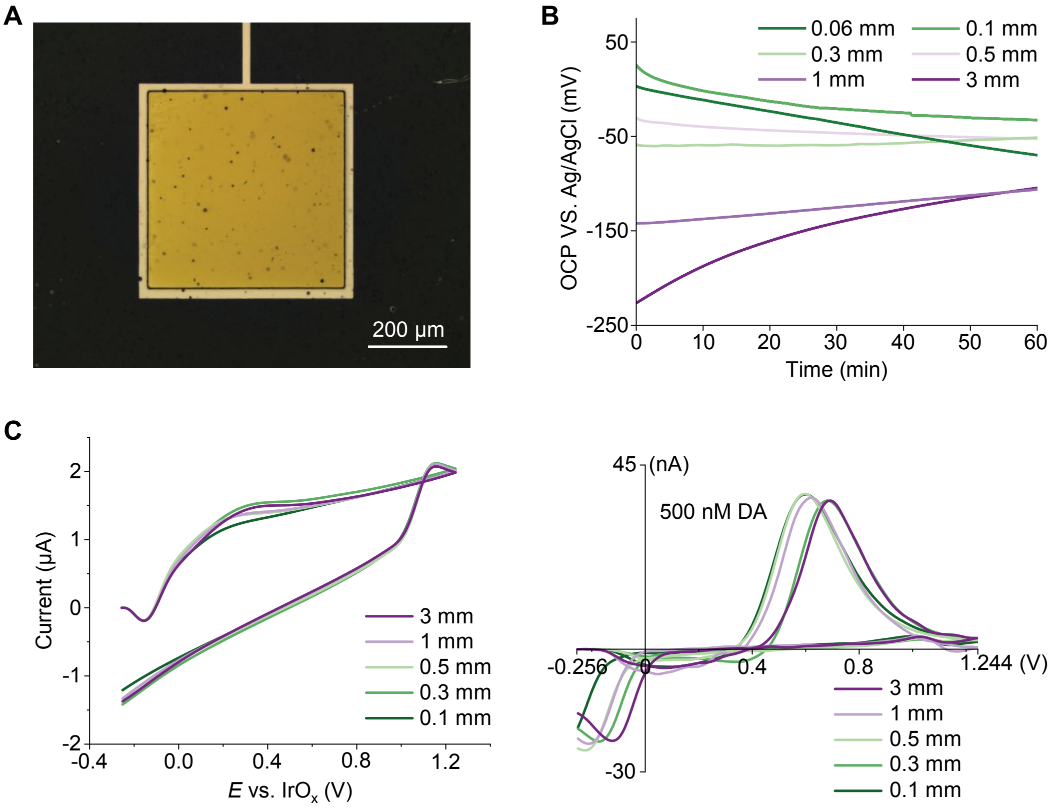

Figure S10. Evaluation of electrodeposited IrO_x as an FSCV reference electrode. (A) Optical microscope image of an IrO_x electrode with a size of 500 μm × 500 μm. (B) Open-circuit potential of IrO_x electrodes with varying sizes, measured against a commercial Ag/AgCl reference electrode (3 M NaCl, BASi, IN, USA). (C) Background current and background-subtracted cyclic voltammograms for 500 nM dopamine, recorded using the IrO_x electrodes as the RE. The IrO_x coating was electroplated at a current density of 0.7 mA/cm² for 130 seconds in a 1.5 g/L $IrCl_4$ solution, following protocols described in [Y. Qiang et al., Adv. Funct. Mater., 27, 1704117 (2017)] and [K. Yamanaka, Jpn. J. Appl. Phys., 28, 632-637 (1989)].

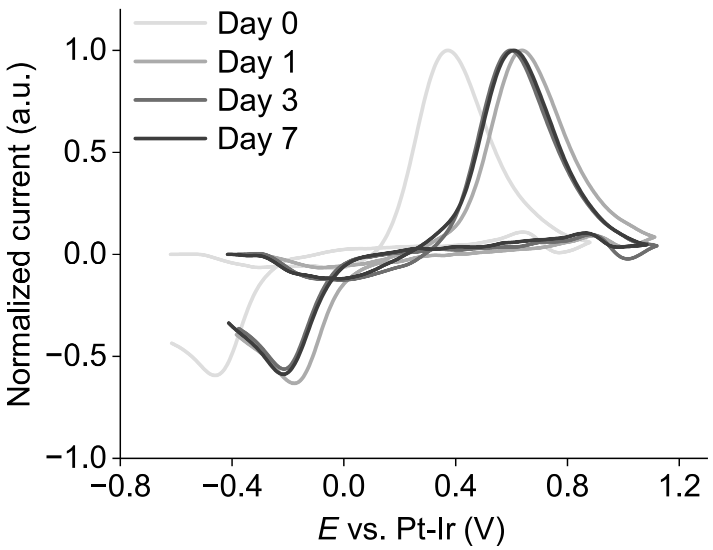

Figure S11. Background-subtracted cyclic voltammograms of 500 nM DA using Pt-Ir as reference electrode after soaking in PBS for the indicated time. Currents were normalized to the peak oxidation current of DA.

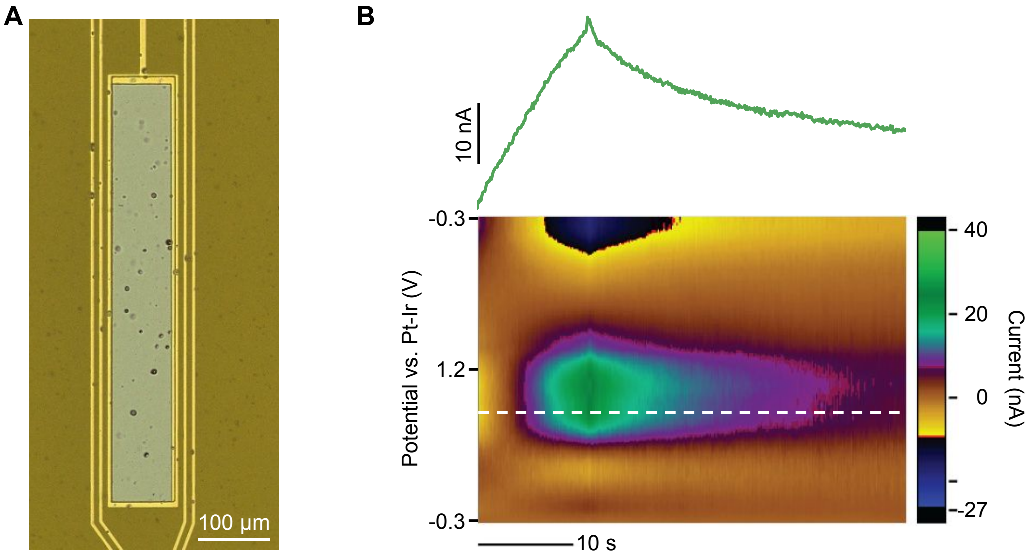

Figure S12. Dopamine sensing in the awake mouse brain using Pt–Ir as the reference electrode. (A) Optical microscope image of a Pt–Ir electrode (90 µm × 600 µm). (B) Background-subtracted color plot (below) and current–time trace (above) at the potential indicated by the dashed line. The dopamine signal was detected in the NAc of head-fixed mouse three days after implantation using a 50 µm × 50 µm carbon-coated microelectrode. The signal was identified as DA signal based on its electrochemical signature and our previous work [Qi, Y. et al., Nat. Commun. 3300 (2025)].

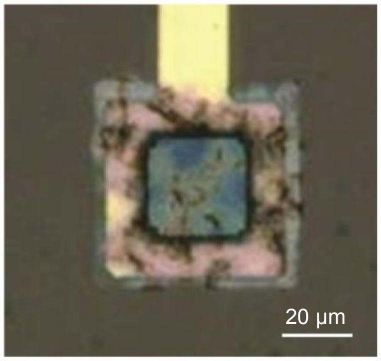

Figure S13. Optical microscope image of a 60 μm × 60 μm carbon-coated microelectrode.
